# The aptamer BT200 effectively inhibits von Willebrand factor (VWF) dependent platelet function after stimulated VWF release by desmopressin or endotoxin

**DOI:** 10.1038/s41598-020-68125-9

**Published:** 2020-07-07

**Authors:** Katarina D. Kovacevic, Nina Buchtele, Christian Schoergenhofer, Ulla Derhaschnig, Georg Gelbenegger, Christine Brostjan, Shuhao Zhu, James C. Gilbert, Bernd Jilma

**Affiliations:** 1 Department of Clinical Pharmacology, Medical University of Vienna, Währinger Gürtel 18-20, 1090 Vienna, Austria; 2 Department of Internal Medicine I, Medical University of Vienna, Vienna, Austria; 3 Division of Vascular Surgery and Surgical Research Laboratories, Department of Surgery, Medical University of Vienna, Vienna, Austria; 4 Guardian Therapeutics, Lexington, MA USA

**Keywords:** Preclinical research, Platelets, Thrombosis

## Abstract

Von Willebrand factor (VWF) plays a major role in arterial thrombosis. Antiplatelet drugs induce only a moderate relative risk reduction after atherothrombosis, and their inhibitory effects are compromised under high shear rates when VWF levels are increased. Therefore, we investigated the ex vivo effects of a third-generation anti-VWF aptamer (BT200) before/after stimulated VWF release. We studied the concentration-effect curves BT200 had on VWF activity, platelet plug formation under high shear rates (PFA), and ristocetin-induced platelet aggregation (Multiplate) before and after desmopressin or endotoxin infusions in healthy volunteers. VWF levels increased > 2.5-fold after desmopressin or endotoxin infusion (p < 0.001) and both agents elevated circulating VWF activity. At baseline, 0.51 µg/ml BT200 reduced VWF activity to 20% of normal, but 2.5-fold higher BT200 levels were required after desmopressin administration (p < 0.001). Similarly, twofold higher BT200 concentrations were needed after endotoxin infusion compared to baseline (p < 0.011). BT200 levels of 0.49 µg/ml prolonged collagen-ADP closure times to > 300 s at baseline, whereas 1.35 µg/ml BT200 were needed 2 h after desmopressin infusion. Similarly, twofold higher BT200 concentrations were necessary to inhibit ristocetin induced aggregation after desmopressin infusion compared to baseline (p < 0.001). Both stimuli elevated plasma VWF levels in a manner representative of thrombotic or pro-inflammatory conditions such as arterial thrombosis. Even under these conditions**,** BT200 potently inhibited VWF activity and VWF-dependent platelet function, but higher BT200 concentrations were required for comparable effects relative to the unstimulated state.

## Introduction

Von Willebrand factor (VWF) is driving the first step and is a key component in platelet thrombus formation when vascular injury occurs under conditions of moderate to high shear force^1,2^. High shear force is commonly found in stenotic arteries and it is known to cause myocardial infarction^3^ or stroke^4,5^. It has been shown previously that plasma levels of VWF are predicting major adverse cardiovascular events in patients with asymptomatic carotid stenosis^6^, as well as in those with acute coronary syndrome^7^. At high shear rates, the main mediator of platelet plug formation is VWF, which is why inhibitors of GPIIb/IIIa, P2Y12 receptor or cyclooxygenase-1 are less potent in conditions where VWF levels are increased^8–10^. Moreover, those antiplatelet drugs typically only produce a limited relative risk reduction in some patients groups such as those with acute coronary syndrome or patients undergoing percutaneous coronary intervention (PCI)^11^. On one end VWF binds to platelets via GpIb, and on the other end it binds to collagen and forms a bridge between platelets and collagen. BT200 is a third-generation aptamer inhibitor of the A1 domain of VWF and prevents VWF from binding to platelet GPIb^12^. BT200 has low nanomolar K_d_ for VWF^12^ and has previously been proven to be effective in human and monkey blood/plasma^12^, as well as in vivo in cynomolgus monkeys^12^. Subcutaneous injection of BT200 in cynomolgus monkeys dose-dependently inhibited VWF and VWF-dependent platelet function. Additionally, BT200 effectively prevented arterial occlusion in an FeCl3-induced thrombosis model in monkeys^12^. Some in vitro data indicate that the effect of the VWF inhibiting nanobody, caplacizumab, differs as a function of the concentration of its target^13^. Therefore, we hypothesized and then specifically examined whether there are shifts in the concentration-effect curves of the VWF inhibitor BT200 before/after stimulated VWF release. We also hypothesized that effects of BT200 will be comparable between healthy volunteers after desmopressin/endotoxin infusion and in patients with chronically elevated VWF levels (carotid stenosis patients) and that BT200′s concentrations and effect will depend on VWF activity.

Endotoxin infusion is a well-established model of inflammation^14^ and coagulation^15^ and leads to VWF release^16^. The vasopressin analogue desmopressin increases circulating VWF levels by stimulating its secretion from the endothelial storage site (Weibel Palade bodies)^17^. Therefore, both endotoxin and desmopressin challenges are useful models in healthy volunteers to test ex-vivo VWF inhibition as would occur in patients with increased VWF plasma levels.

## Methods

We used samples from two different trials which were approved by the Ethics Committee of the Medical University of Vienna, all methods were performed in accordance with the relevant guidelines and regulations**;** all subjects provided written informed consent. Both studies were randomized, placebo-controlled in a cross-over design. However, in the placebo period we only analyzed data from 6 subjects (4 from desmopressin trial and 2 from endotoxin trial) in total due to the consistency of results and the lack of VWF-dependent shifts in the concentration-effect curves.

### Desmopressin trial

Twenty healthy volunteers aged between 18 and 65 were included in the desmopressin trial, which also investigated the half-life of other Weibel Palade constituents^18^. On the first day of the study, subjects were randomized to 1-desamino-8-d-arginin-vasopressin (DDAVP, desmopressin) or placebo in a double- blinded manner. Two weeks after the first study day, subjects received the other drug (either desmopressin or placebo). Therefore, all subjects received both desmopressin and placebo. However, after analysis of the first 4 subjects who received placebo, we focused on the analysis of the desmopressin period (16 subjects), because results before/after placebo were consistent. Desmopressin (Octostim, Uniprix) was diluted in 50 ml 0.9% NaCl and infused at a dose of 0.3 μg/kg over 30 min. Placebo (0.9% NaCl) was infused at the same rate over the same time period. Samples from healthy volunteers were drawn via a butterfly needle into tubes containing hirudin or 3.8% citrate/hirudin at 0 min, 15 min, 30 min, 60 min, 90 min, 2, 3, 4, 6, 8, 24 h after desmopressin/placebo infusion. VWF antigen was measured at all time points and VWF activity, impedance aggregometry and platelet function tests were done at 0 h and 2 h after desmopressin/placebo infusion. After performing tests with whole blood (impedance aggregometry and platelet function tests), samples were centrifuged at 2000*g* for 10 min and stored at − 80 °C until analysis.

### LPS trial

The study design of the lipopolysaccharide (LPS, endotoxin) trial was recently published^19^. Twenty healthy volunteers participated in the LPS trial. On the first day 16 of them received LPS and 4 of them received placebo. We performed analyses of 16 subjects who received LPS and 2 who received placebo. Blood was drawn from healthy volunteers with a butterfly needle at − 1 h, 0, 1, 2, 4, 6 and 24 h after placebo/LPS infusion into tubes containing 3.8% citrate. VWF antigen was measured at all time points. Concentration- effect curves of BT200 on VWF activity, impedance aggregometry and platelet function tests were performed at 0 h and 4 h after placebo/LPS stimulation (2 ng/kg bodyweight bolus). Samples were centrifuged at 2000*g* for 10 min and plasma was stored at − 80 °C until VWF activity and antigen analysis were performed.

### ICARAS study

As previously published, 811 patients with carotid stenosis were included in this study^6^. We stratified plasma from 30 patients from this study into pools according to their VWF activity levels (Pool 1 < 75%, pool 2 > 75%, pool 3 > 200%, pool 4 > 300%, pool 5 > 400%, pool 6 > 500%). Pools were spiked with 8 different BT200 concentrations and VWF activity was measured.

### Measurement of inhibitory effects of BT200 on platelet function in the desmopressin trial

#### Platelet function analyzer 100 (PFA-100)

The effect of BT200 on VWF-mediated, shear-dependent platelet function was examined with the Platelet Function Analyzer PFA-100 (Dade Behring). The PFA-100 quantifies the rate at which a platelet “plug” can form under shear stress; the time needed for the aperture occlusion is reported as closure time (CT). Even under normal conditions (i.e. in healthy volunteers), there is a high degree of correlation between VWF and CT values particularly when measured repetitively to minimize biologic and analytical variability^20^. The primary adhesion process in the PFA occurs through VWF/GpIb interaction as demonstrated by inhibitors of VWF (the platelet plug formation also involves the interaction of VWF with GPIIb/IIIa)^8^. We measured collagen adenosine diphosphate (CADP-CT) induced closure time in whole blood samples anti-coagulated with 3.8% sodium citrate, and incubated with eight increasing BT200 concentrations in a water bath (37 °C) for 15 min prior to analysis. The instrument records the time until aperture occlusion by the formation of a platelet plug [i.e., the Closure Time (CT)] up to a maximum of 300 s^21^. All measurements were done within 1 h of blood sampling.

#### Impedance aggregometry (multiple electrode aggregometry)

Fresh blood was anti-coagulated with hirudin and after > 30 min it was incubated for 15 min at 37 °C in a water bath with 8 different BT200 concentrations (0–9 µg/ml: approximately equivalent to 0–15 µg/ml in plasma). Platelet aggregation was measured using a commercially available impedance aggregometer (Multiplate Roche)^22^ using a 5-channel device with disposable test cells and a dual-sensor unit. Ristocetin 0.77 mg/mL was used to stimulate platelet aggregation by VWF co-activation. Multiplate continuously records platelet aggregation; the increase of impedance by the attachment of platelets onto the Multiplate sensors is transformed into arbitrary aggregation units (U) and plotted against time. The most important parameter calculated is the area under the aggregation curve (AUC); normal values for AUC are 44–176 U^23^.

In order to investigate whether VWF multimer profiles would influence BT200 effects, we compared samples from two healthy individuals with normal VWF levels and presumed normal multimers with samples from a patient suffering from congenital thrombotic thrombocytopenic purpura due to ADAMTS-13 deficiency^24,25^ with increased levels of ultra large VWF multimers and this allowed us to measure BT200 effects in VWF dependent platelet function.

### Measurement of ex vivo inhibitory effect of BT200 on VWF activity

#### VWF activity

The amount of active VWF in human plasma and the ex vivo inhibitory effect of BT200 on VWF activity was evaluated with a commercially available ELISA kit (REAADS VWF Activity ELISA Test Kit, Corgenix, Inc, Westminster, Colo)^12^. A monoclonal antibody specific for the A1 domain of VWF is coated to the 96-microwell plates^26,27^. Diluted plasma samples are incubated in the wells, which are washed and bound antigen is detected by a horseradish peroxidase conjugated anti-human VWF detection antibody. BT200 competes with antibody binding in the ELISA assay^12^. As both BT200 and the ELISA antibody have the same target (A1 domain of VWF), in the presence of BT200 less A1 domains are available for the ELISA antibody, which allows measurement of the inhibitory effect of BT200 on VWF activity.

Stored plasma samples were thawed and incubated for 15 min in a water bath at 37 °C with 8 different concentrations of BT200 (0–15 µg/ml) and the assay was run at a dilution of 1:3 (which is different from the 1:21 suggested by the kit manufacturer.) This protocol change relates to the influence of the greater dilution on the balance between free and bound aptamer by washing the aptamer off its target. The standard curve has also been adapted according to the new dilution. This dilution step is done before the samples are pipetted into the ELISA plate. The median coefficient of variation for intra-assay variability was < 5% and the inter-assay variability was < 12%.

In order to further validate our results, similar BT200 spiking experiments were performed in samples obtained from patients with carotid stenosis, where VWF levels predict major adverse cardiovascular events^6^. Samples from 811 patients were pooled into 5 groups according to their VWF levels (VWF activity < 75%; 75–199%; > 200%; > 300%; > 400%; > 500%) and 8 different BT200 concentrations were spiked into each pool.

#### VWF antigen levels

For measuring VWF antigen levels a commercially available ELISA kit was applied (REAADS VWF Antigen ELISA Test Kit, Corgenix, Inc, Westminster, Colo). Samples from all available time points were analyzed for both the DDAVP (0 min, 15 min, 30 min, 60 min, 90 min, 2 h, 3 h, 4 h, 6 h, 8 h, 24 h) and the LPS trial (− 1 h, 0 h, 1 h, 2 h, 4 h, 6 h, 24 h). The VWF antigen ELISA was performed following the manufacturer’s instructions^6^.

### Statistical methods

All data are expressed as means and 95% confidence interval (95% CI) unless otherwise stated. Statistical tests included the Friedman ANOVA for time courses and the Wilcoxon signed ranks test for post-hoc comparisons. A 2-tailed P value of less than 0.05 was considered significant. No correction for multiple tests was performed because all tests are VWF dependent. Target concentrations of interest were 20% of normal VWF activity^28^. The platelet function analyzer is used as a screening method for measuring VWF activity^21^, target concentrations for the PFA were the ones that maximally prolonged CADP-CT (> 300 s). Target concentrations for Multiplate were the ones which lowered AUC values to < 20 U, because 25 U were seen in patients with von Willebrand disease (VWD) type 2 who had VWF levels of 24%, and 13 U and 9 U were seen in patients with VWD type 3 without therapy, these had VWF levels of 12% and 0% respectively^29^.

## Results

### Desmopressin and LPS increase circulating VWF antigen levels

The mean VWF antigen level in the desmopressin trial was 97% (95% CI 83–111) at baseline and increased 2.9-fold 90 min after the start of desmopressin infusion [284% (95% CI 250–318); p < 0.001]. The mean VWF antigen level was 101% (95% CI 86–116) at baseline and increased 2.5-fold 4 h after LPS infusion [245% (95% CI 196–294); p < 0.001]. As expected, VWF antigen did not change significantly after placebo infusion in both trials.

### VWF activity is inhibited by BT200

**Figure 1:**
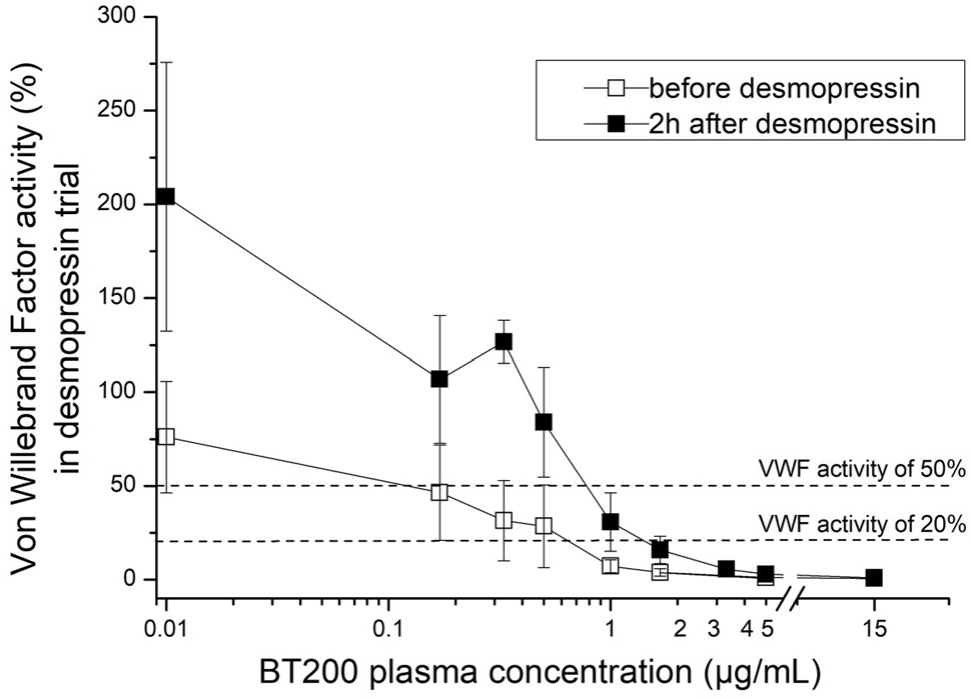
Concentration effect curves of the von Willebrand factor (VWF) inhibiting aptamer BT200 on VWF activity before (white squares) and 2 h after desmopressin infusion (black squares) (16 healthy volunteers). BT200 was spiked ex vivo into citrated plasma with 8 different concentrations. The difference between BT200 concentration needed to supress VWF activity to < 20% of normal was significant before and after desmopressin (p < 0.001). Baseline (0 µg/ml) is depicted as 0.01 to improve visualisation in all figures with log scales. Data are presented as means ± 95% CI (*VWF* Von Willebrand factor).

**Figure 2:**
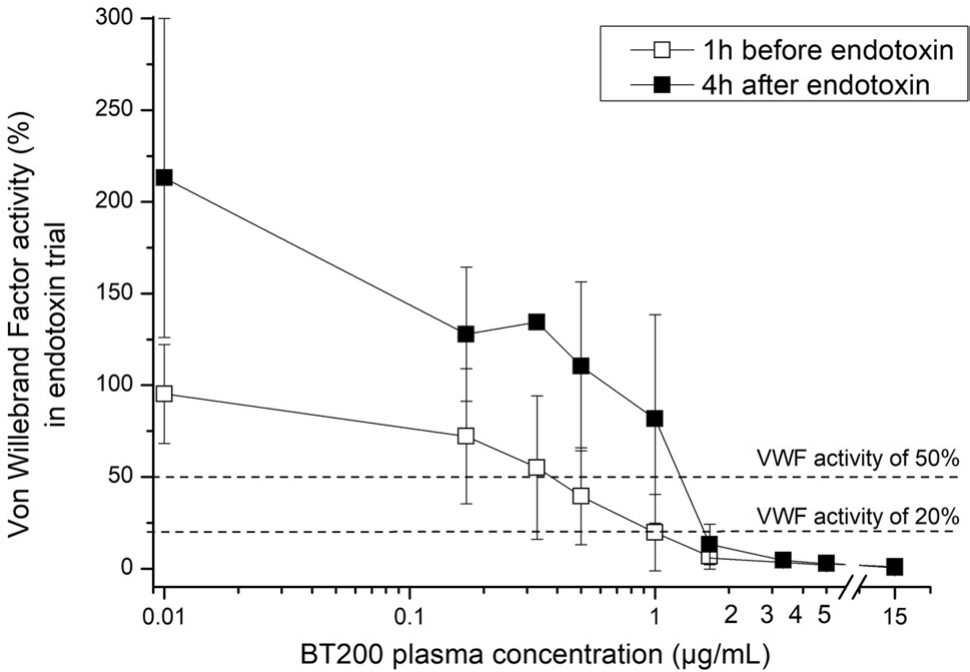
Concentration effect curves of the von Willebrand factor inhibiting aptamer BT200 on VWF activity before (white squares) and 4 h after infusion of 2 ng/kg endotoxin (black squares) (17 healthy volunteers). BT200 was spiked ex vivo into citrated plasma with 8 different concentrations. The difference between BT200 concentration needed to supress VWF activity to < 20% of normal was significant before/after endotoxin (p < 0.001). Baseline (0 µg/ml) is depicted as 0.01 to improve visualisation in all figures with log scales. Data are presented as means ± 95% CI (*VWF* Von Willebrand factor).

In the desmopressin trial, the mean baseline VWF activity was 76% (95% CI 61–91) and it increased 2.7-fold to 204% (95% CI 169–239) at 2 h after desmopressin challenge (p < 0.001). Higher concentrations of BT200 were needed after desmopressin infusion to inhibit VWF activity (Fig. 1). At baseline, plasma concentrations 0.51 µg/ml of BT200 reduced VWF activity to < 20% of normal. However, 2.6-fold higher BT200 concentrations (1.31 µg/ml) were required to achieve similar inhibition of VWF activity two hours after desmopressin infusion (p = 0.001). The shift in the concentration-effect relationship is also presented visually in Suppl. Fig S1. In the LPS trial, the mean baseline VWF activity was 95% (95% CI 81–109) which increased 2.2-fold to 213% (95% CI 169–257; p < 0.001) 4 h after endotoxin challenge. BT200 concentrations of 0.87 µg/ml were required to reduce VWF activity to < 20% of normal at baseline and 1.55 µg/ml of BT200 4 h after endotoxin infusion (p = 0.011) (Fig. 2). Similar results were obtained with samples from patients with carotid stenosis: 0.5 µg/ml of BT200 was needed to inhibit VWF activity to < 20% in the pool with the lowest VWF activity (< 75%) and 5 µg/ml of BT200 was necessary in the group with the highest VWF levels (> 500%) (data not shown).

After placebo infusion, no changes were noticed in the concentration-effect relationship when comparing values at baseline and after infusion of desmopressin or endotoxin (Suppl. Figs. S2, S3).

We did a sensitivity analysis where we stratified subjects from both trials into two groups, according to their VWF activity, one with VWF activity < 75% and the other with VWF activity > 75% and we compared the difference in BT200 concentrations required to decrease VWF activity to < 20% of normal. There was a significant difference in the required BT200 concentrations (p < 0.001) between these two groups, even though the VWF multimer profile should be comparable (Supp. Fig. S7).

To examine whether this observation after acute VWF release also holds true for patients with chronically elevated VWF levels, we generated 6 plasma pools stratified according to VWF activity with samples from the previously published ICARAS study^6^; higher BT200 concentrations were needed in pools with higher VWF activity to decrease VWF activity to < 20% of normal; ranging from 0.4 to 5 µg/ml for 1st to 6th pool respectively (Supp. Fig. S8).

### Ex vivo inhibitory effect of BT200 on platelet function after desmopressin infusion

**Figure 3:**
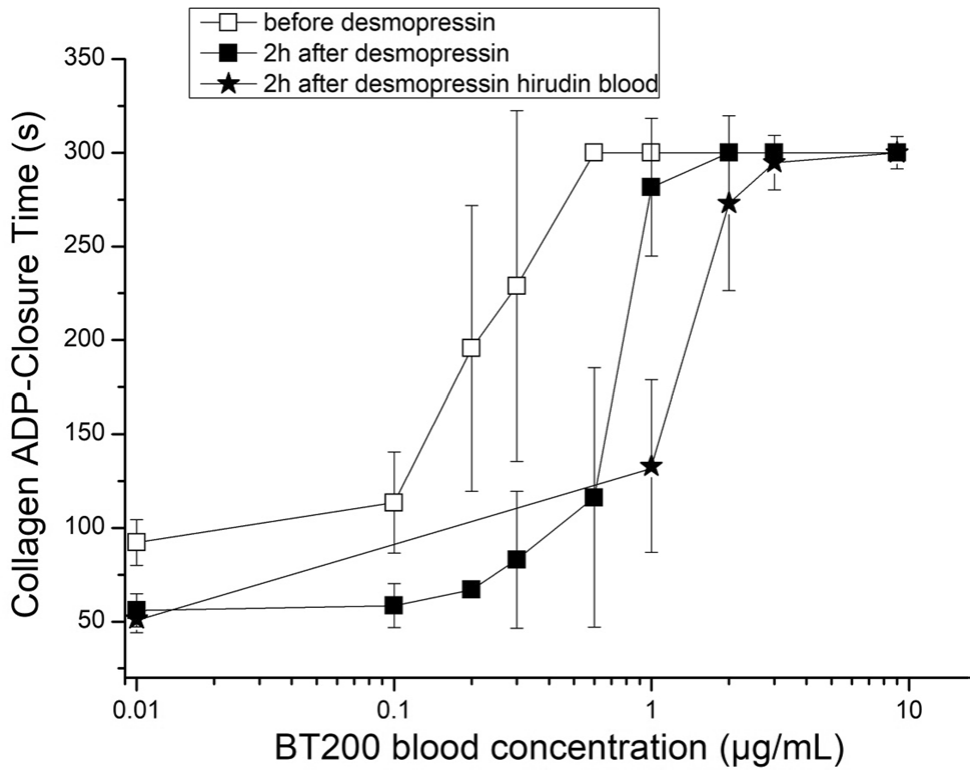
Concentration effect curves of BT200 on platelet plug formation under high shear rates before and after desmopressin infusion. Blood samples were anticoagulated with either citrate or hirudin and spiked with BT200 at 8 different concentrations (16 healthy volunteers). Collagen adenosine diphosphate closure time (CADP-CT) was measured by the platelet function analyzer PFA-100. Note the shorter CADP-CT values after desmopressin. The difference between BT200 concentrations needed to maximally prolong CADP-CT was significant before and after desmopressin in citrate blood (p < 0.001). Data are presented as means ± 95% CI. The instrument records the time until aperture occlusion by the formation of a platelet plug (i.e., the Closure Time) up to a maximum of 300 s.

*PFA-100:* desmopressin infusion shortened the mean collagen adenosine diphosphate closure time (CADP-CT) from 92 s (95% CI 86–98 s) at baseline to 56 s (95% CI 52–60 s; p < 0.001) 2 h after infusion. Average levels of 0.49 µg/ml BT200 were needed to maximally prolong CADP-CT to > 300 s at baseline and more than twofold higher concentrations of BT200 (1.35 µg/ml) were required 2 h after desmopressin challenge (p = 0.001). Significantly higher concentrations of BT200 (3.19 µg/ml) were needed to prolong CADP-CT in blood anticoagulated with hirudin as opposed to sodium citrate (Fig. 3) (p = 0.002).

#### Whole blood aggregometry

**Figure 4:**
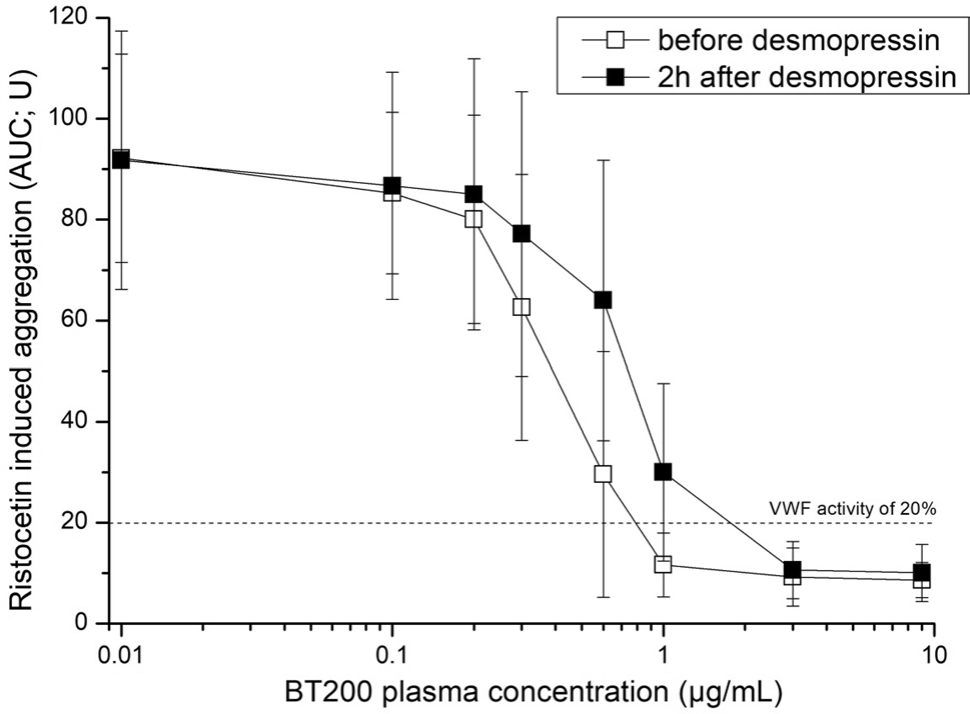
Concentration effect curves of the von Willebrand factor inhibiting aptamer BT200 on ristocetin induced whole blood aggregation before and after desmopressin infusion. Hirudin anticoagulated blood was spiked ex vivo with 8 different BT200 concentrations and aggregation was measured with the impedance aggregometer Multiplate. The difference between BT200 concentration needed to inhibit ristocetin induced aggregation to < 20 U was before/after desmopressin (p < 0.001). Data are presented as means ± 95% CI.

The mean baseline AUC value of ristocetin induced aggregation was 92 U (95% CI 82–102 U), which was unaffected by desmopressin infusion (p = 0.96). Concentrations of 0.69 µg/ml of BT200 were required to reduce ristocetin-induced aggregation to less than 20 U at baseline, and twofold higher concentrations (1.31 µg/ml) were needed 2 h after desmopressin infusion (p = 0.001) (Fig. 4). Placebo did not alter the concentration-effect curves of BT200 in any of the three assays (Suppl. Figs. S2, S4 and S5).

Fluctuations of platelet counts corresponded to the fluctuations in hemoglobin and there were no significant differences between the desmopressin and placebo groups (Suppl. Fig. S6).

To explore whether high molecular weight VWF multimers could be a major contributor, we examined the inhibition of VWF dependent platelet function in a patient suffering from congenital thrombotic thrombocytopenic. Similar BT200 concentrations were needed to maximally prolong CADP-CT and to inhibit aggregation to < 20 U in the patient compared to samples from 2 healthy individuals when both were measured with PFA100 and Multiplate (Supp. Fig. S9).

## Discussion

While VWF presents an obvious and attractive target to prevent or treat arterial thrombosis, VWF inhibitors have only relatively recently reached the stage of clinical development, and then only in the form of macromolecules such as the monoclonal antibody AJW-200^30^, the nanobody caplacizumab used in thrombotic thrombocytopenic purpura^31–33^ and the aptamer ARC1779 used in various indications^34–38^. Proof of concept for VWF inhibition in patients was first established with an anti-VWF aptamer conjugated to a 20 kDa polyethylene glycol molecule (ARC1779). ARC1779 was generally safe and effective, but the limited half-life of approximately 2 h meant it was only suitable if administered as a continuous infusion^24,34,35,39–42^. As stated in a recent review^36^, improvement in the half-life is seen with higher PEG moieties, which has led to a considerable half-life extension to ~ 66 h for ARC15105^37^, which contains a 40 kDa PEG residue similar to BT200. Inhibiting VWF improves the outcome in patients with thrombotic thrombocytopenic purpura^32,33^, but it could also offer potential benefits for patients suffering from stroke^38^, myocardial infarction^3^, or other thrombotic diseases such as sickle cell disease^43^.

The major finding of our study is that BT200 effects are target-concentration dependent, which means that higher concentrations of the VWF inhibitor are needed to produce the same effect in the presence of higher levels of circulating VWF. Taken together with preliminary data on other VWF inhibitors, these results likely indicate a class effect of VWF inhibitors^13,30,44^. We decided to look back at our results obtained for ARC1779 across various populations^45, 46^, which confirmed the target concentration-dependent inhibition of VWF by BT200 seen in the current prospective study (data not shown). Similarly, we confirmed a target concentration-dependent inhibition of VWF in samples from patients with carotid atherosclerosis. What could be a potential explanation for the target concentration-dependent effect? IC50 values in our study are quite similar**.** We have defined the target concentration of BT200 as the one lowering VWF levels to < 20% of normal. In contrast to more tightly regulated coagulation factor levels such as prothrombin (1–2 μM^47^) or factor × (0.1 μg/ml^48^), VWF concentrations are more dispersed (larger intersubject variability). If one subject has a starting level of 200% VWF activity, an IC90 is required to obtain 20% residual activity, whereas if the starting level is 80% an IC75 would be sufficient.

The required inhibitory concentrations were similar between different assays showing internal consistency. The increase in VWF antigen levels and VWF activity induced by endotoxemia or desmopressin infusion was consistent with previous trials^9,20,49,50^, as was the enhanced platelet plug formation under high shear rates^9,20^, demonstrating the external validity of the results. Plasma VWF levels > 200% are typically found in patients with myocardial infarction or stroke at high risk of recurrent events and death^3,7,51^. Therefore, the infusion of desmopressin and endotoxin provided appropriate human models to simulate the effects of BT200 under clinically relevant conditions of increased plasma VWF.

Notably, the effects of P2Y12-receptor and GPIIb/IIIa inhibitors or aspirin, particularly when measured under pathophysiological relevant high shear rates, are compromised in conditions where VWF levels are increased^8–10^. For example, VWF release during endotoxemia partly antagonized the inhibitory effect of prasugrel as measured with the PFA-100 system^10^. Similar results were obtained for aspirin or nitric oxide coupled aspirin^52^. Furthermore, the desmopressin induced VWF release accelerated the normalization of the prolonged CADP-CT by GPIIb/IIIa inhibitors (plus l-aspirin)^9^. This means that the efficacy of GPIIb/IIIa inhibitors, similar to aspirin or prasugrel, will be limited in cases of increased circulating VWF. Similar to previous observations, the apparent platelet inhibition by aspirin was mitigated in the presence of physiological calcium concentrations^9^; anticoagulation of samples with hirudin instead of citrate required higher BT200 concentrations to produce the same effect as those found in citrated blood. This is also in agreement with a study using ARC1779^45^ where threefold higher concentrations were required in hirudin anticoagulated samples. This is likely due to the fact that VWF requires calcium to facilitate platelet adhesion^53^ and hirudin, unlike citrate, does not complex calcium ions^45^. When comparing BT200 with the anti-VWF nanobody caplacizumab, which has already been authorized for acquired thrombotic thrombocytopenic purpura^32,33,54^, a potential benefit of BT200 would be a longer half-life, which was > 100 h in non-human primates^12^ vs. 5–36 h for caplacizumab^55^. This means that BT200 is likely suited to being administered once a week as a subcutaneous injection, instead of the daily injections required with caplacizumab. As traumatic bleedings may occur, and emergency operations may be necessary, a reversal agent called BT101 has been developed, which effectively inhibited BT200 in non-human primates^56^. As yet, no reversal agent is available for caplacizumab, meaning that treatment of such acute events under caplacizumab have to managed with VWF concentrates, recombinant VWF or desmopressin alone or in combination. Additionally, the manufacturing process of aptamers is easier when compared to antibody production^57^ and the aptamers are heat stable.

Interestingly, desmopressin did not enhance ristocetin induced platelet aggregation in whole blood in the absence of BT200. One possible explanation is that the test system is insufficiently sensitive to elevated VWF levels, which is in contrast to the shortening of closure times in the PFA-100 when VWF levels increase^9,58^. However, higher BT200 concentrations were required after desmopressin-stimulated VWF release to produce the same inhibition of platelet aggregation when compared with baseline. Therefore, the test system appears to be more sensitive to changes in the low VWF activity range; indeed this is what the test is intended for in routine diagnostic laboratory use, and it reaches a plateau when VWF levels exceed 50% (data not shown).

The suitability of using desmopressin and endotoxin as a surrogate for the conditions where VWF is chronically elevated is supported by demonstrating similar effects of BT200 in both pooled plasma from patients suffering from carotid stenosis and in healthy volunteers after desmopressin/endotoxin infusion. Under all conditions, BT200 concentrations were target concentration-dependent.

### Limitations

Concentration effect curves were only established ex vivo. However, as BT200 inhibits a plasma protein, concentrations may be directly extrapolated to circulating VWF levels. As concentrations of VWF increased, we deliberately chose not to calculate IC50 or IC90 values but were interested in which concentrations suppress VWF to about 20% of normal values. The exact level of VWF inhibition needed to prevent arterial thrombosis is unknown. The anti-VWF nanobody caplacizumab exerts almost complete inhibition of VWF^59^ which benefits patients with thrombotic thrombocytopenic purpura. Based on epidemiologic data from patients with von Willebrand disease which showed approximately 40–60% fewer arterial thrombotic events in patients with VWF activity of about 25%^28^, we assumed that decreasing VWF activity to 20% or less is of therapeutic interest. We used citrate as an anticoagulant for the VWF activity assay and the PFA assay, which was in accordance with the manufacturer’s instructions. This may underestimate the required BT200 plasma concentrations. However, we additionally performed PFA analysis with hirudin blood and also performed whole blood aggregation in hirudin, and this mitigates this limitation. Although we have not analyzed VWF multimers, it has previously been shown that both desmopressin and LPS increase levels of high molecular weight VWF multimers in blood^9,16,58^. Even at baseline before the challenges were performed, a stage at which healthy volunteers likely have comparable patterns of multimers, BT200 concentrations needed to suppress VWF activity to < 20% of normal were dependent on VWF activity. Additionally, a spiking experiment with BT200 showed comparable concentration-effect curves on closure time and ristocetin induced aggregation in healthy volunteers and in a patient suffering from congenital thrombotic thrombocytopenic purpura, a condition where patients have ultra large VWF multimers and enhanced platelet aggregation^24^. This small experiment is in agreement with a previous study which showed comparable IC90 values for the anti-VWF aptamer ARC1779 in VWF dependent platelet function tests in healthy volunteers and thrombotic thrombocytopenic purpura patients^46^. Additionally, ARC1779 successfully inhibited VWF activity and platelet function in vivo^24^ in patients with thrombotic thrombocytopenic purpura. These analyses support the concept that inhibitory concentrations more likely depend on the mass of circulating VWF than on its multimeric distribution.

## Conclusion

Our trials indicate that the effects of the BT200 anti-VWF aptamer are target concentration-dependent, which seems to be a class effect. This is important for dose-finding and translation of data from healthy subjects to patients with elevated VWF levels and lays the foundation for defining and predicting dose ranges in patients at risk for arterial thrombosis. Even under conditions of stimulated VWF release**,** BT200 potently inhibits VWF activity and VWF-dependent platelet function. This is in contrast to conventional platelet inhibitors whose effects are compromised by elevated VWF under high shear rates.

## Supplementary information

Supplementary figures
