# Supplementary material for: The aptamer BT200 effectively inhibits von Willebrand factor (VWF) dependent platelet function after stimulated VWF release by desmopressin or endotoxin
Source: Sci Rep. 2020 Jul 7;10:11180. doi: 10.1038/s41598-020-68125-9 (PMC7341806; doi:10.1038/s41598-020-68125-9)
Supplement: Supplementary file 1 — Supplementary figures [file 41598_2020_68125_MOESM1_ESM.docx]

**The aptamer BT200 effectively inhibits von Willebrand factor (VWF) dependent platelet function after stimulated VWF release by desmopressin or endotoxin**

Katarina D. Kovacevic^1^, Nina Buchtele^2^, Christian Schoergenhofer^1^, Ulla Derhaschnig^1^, Georg Gelbenegger^1^, Christine Brostjan^3^, Shuhao Zhu^4^, James C Gilbert^4^, Bernd Jilma^1^

^1^Department of Clinical Pharmacology

^2^Department of Internal Medicine I,

^3^Division of Vascular Surgery and Surgical Research Laboratories, Department of Surgery, Medical University of Vienna, Vienna, Austria

^4^Guardian Therapeutics, Lexington, MA, USA

**Number of figures: 4, Number of references: 48**, **Supplemental data: 7 figures**

**Format:** Original article

Corresponding author:
Bernd Jilma, MD
Department of Clinical Pharmacology, Medical University of Vienna
Währinger Gürtel 18-20, 1090 Vienna, Austria
Tel.: +43 1 40400 29810
Fax: +43 1 40400 29980
Email: [bernd.jilma@meduniwien.ac.at](mailto:bernd.jilma@meduniwien.ac.at)

**Supplementary Figures**


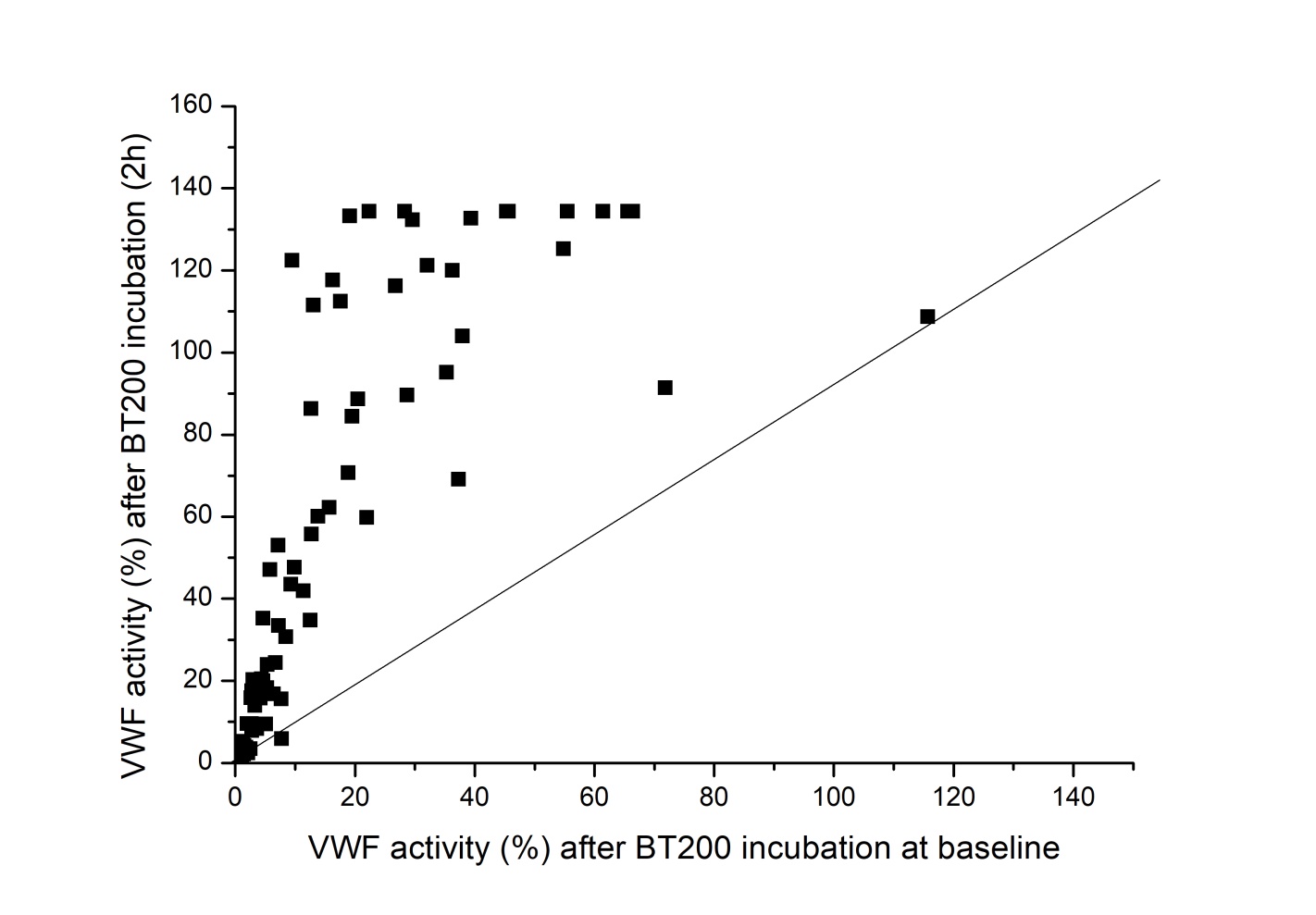


Supplementary Figure S1. The curvilinear relationship between Von Willebrand Factor (VWF) activity after BT200 incubation before and 2 hours after desmopressin infusion. Data points are from concentration-effect curves. All dots would be expected on the median line if there was no shift in the concentration-effect curve.


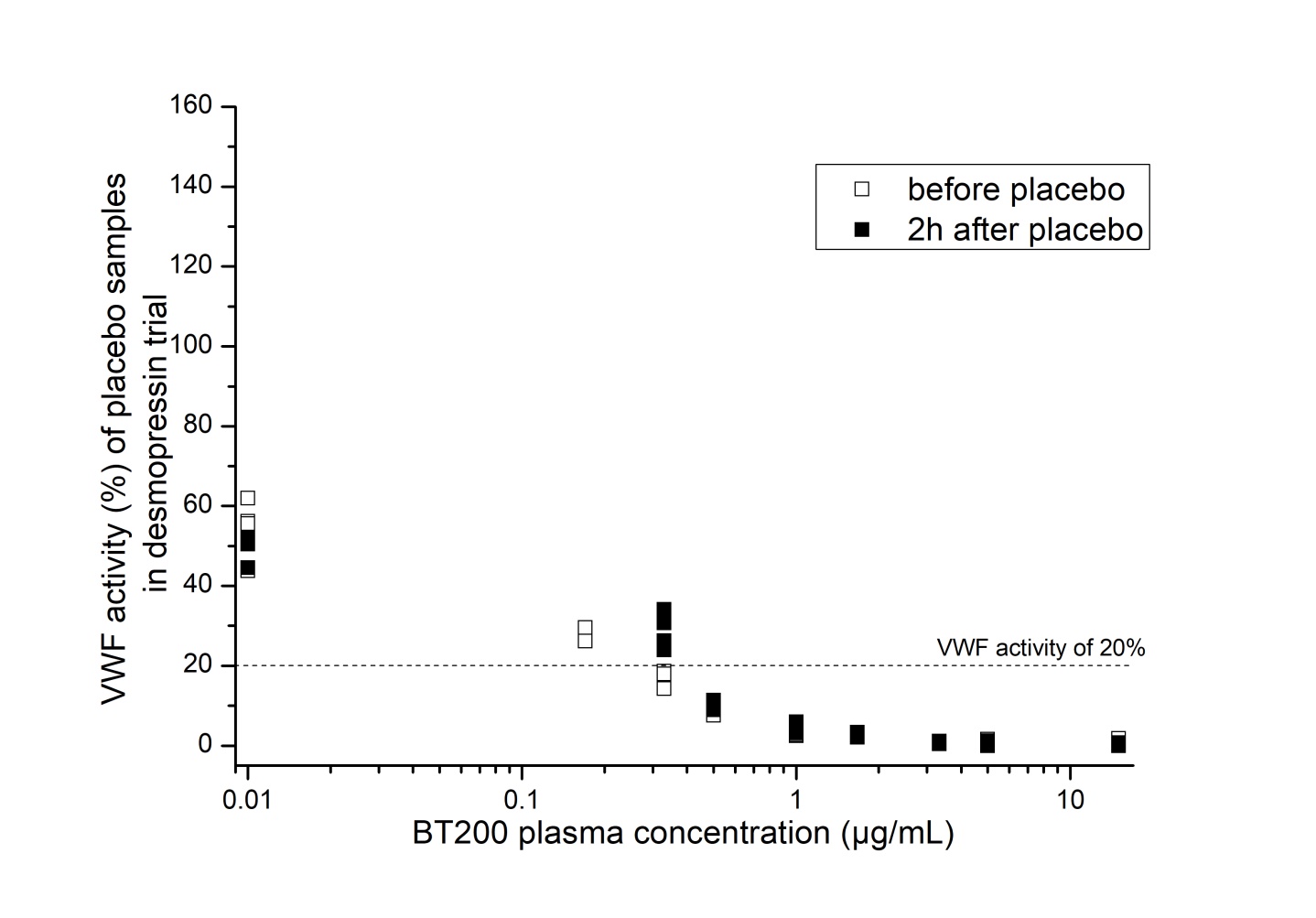


Supplementary Figure S2. Mean concentration-effect curve of BT200 on VWF activity plasma samples obtained from healthy volunteers receiving only placebo in the desmopressin trial (measured in 4 healthy volunteers). Eight different BT200 concentrations were spiked into citrated plasma. The difference between BT200 concentration needed to supress VWF activity to <20% of normal was not significant before and after placebo (p=0.29). Baseline (0µg/ml) is depicted as 0.01 to improve visualisation in all figures with log scales. (VWF-Von Willebrand Factor)


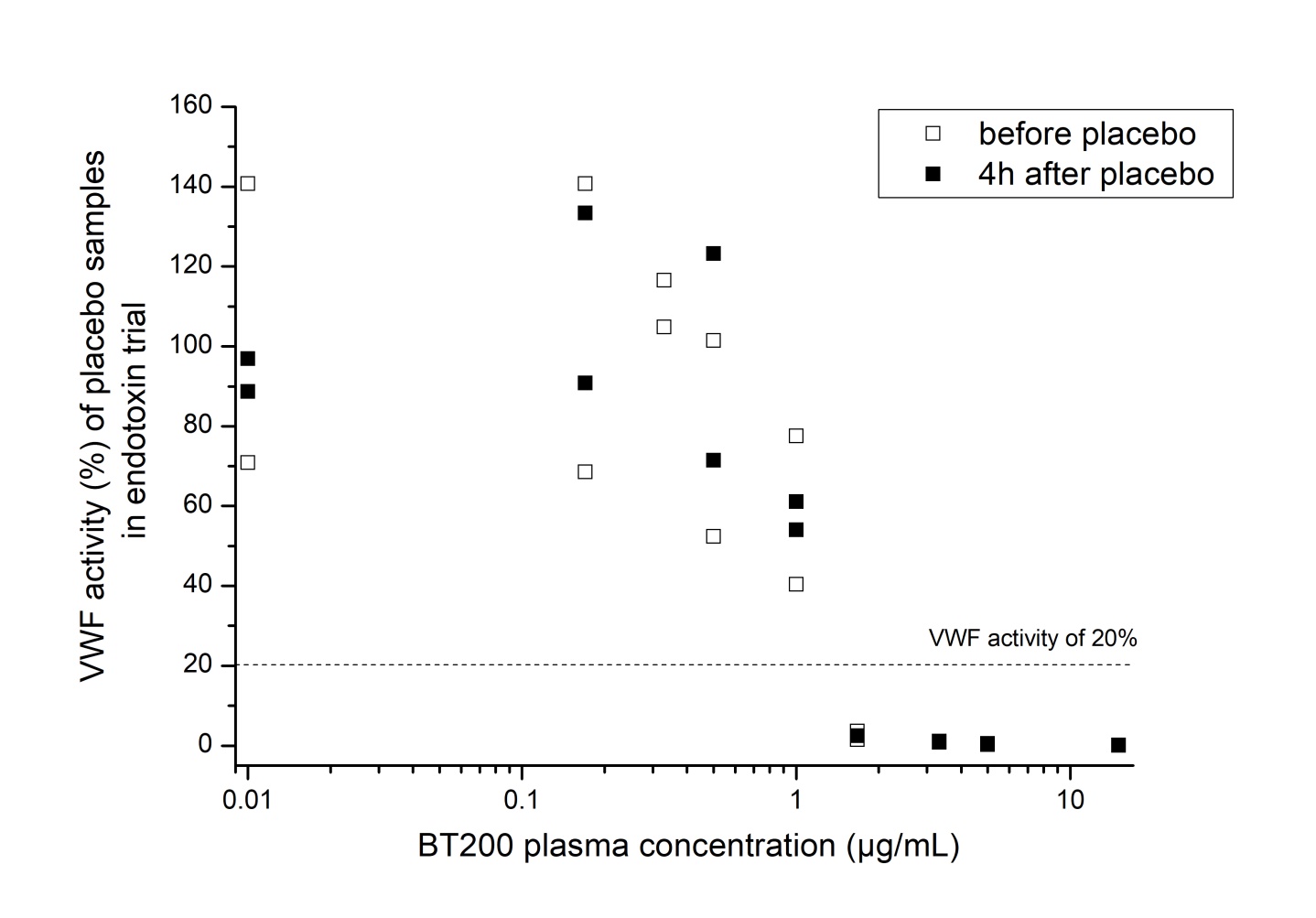


Supplementary Figure S3. Mean concentration-effect curve of BT200 on VWF activity plasma samples obtained from healthy volunteers receiving only placebo in the endotoxin trial (measured in 2 healthy volunteers). Eight different BT200 concentrations were spiked into citrated plasma. The difference between BT200 concentration needed to supress VWF activity to <20% of normal was not significant before and after placebo (p=0.93). Baseline (0µg/ml) is depicted as 0.01 to improve visualisation in all figures with log scales. (VWF-Von Willebrand Factor)


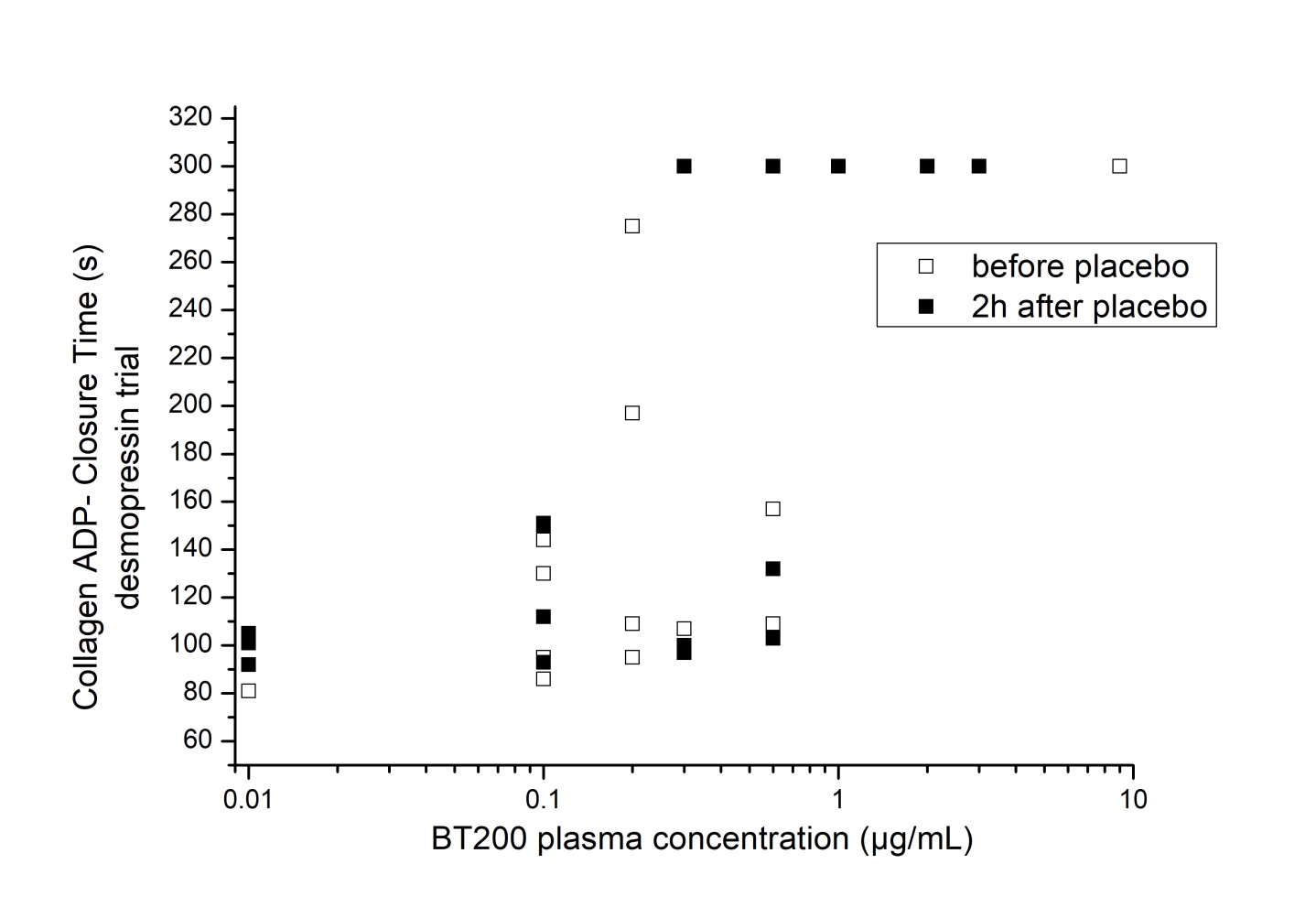


Supplementary Figure S4. Mean concentration-effect curve of BT200 on platelet plug formation under high shear rates before and 2 hours after placebo infusion in the desmopressin trial (4 healthy volunteers). Citrated blood was spiked ex vivo with 8 different concentrations of the von Willebrand Factor inhibiting aptamer BT200. The difference between BT200 concentration needed to maximally prolong CADP-CT was not significant before and after placebo (p=0.51). Baseline (0µg/ml) is depicted as 0.01 to improve visualisation in all figures with log scales.


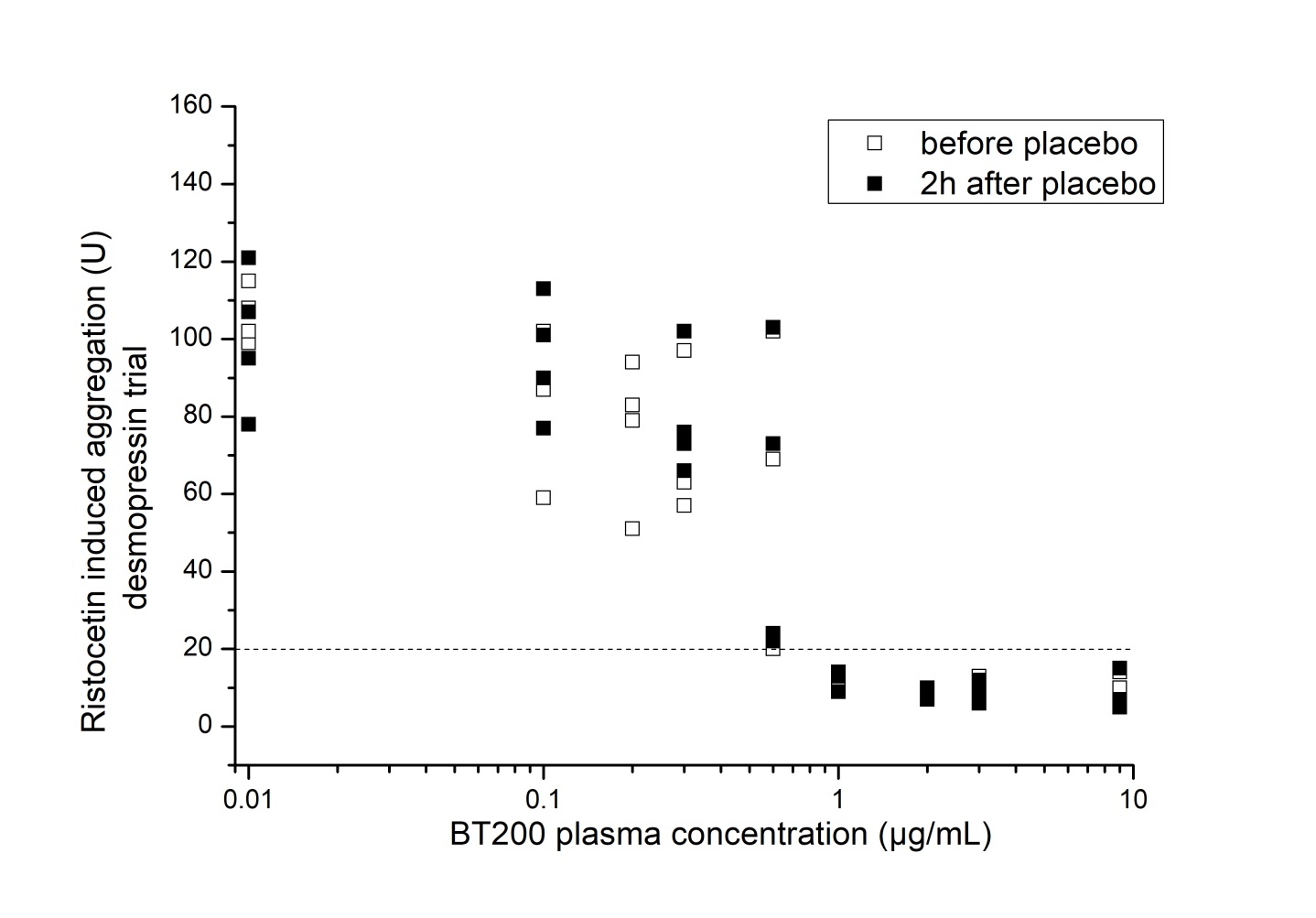


Supplementary Figure S5. Mean values of ristocetin induced aggregation before and 2 hours after placebo infusion in the desmopressin trial (4 healthy volunteers). Blood samples were spiked ex vivo with 8 different concentrations of the von Willebrand Factor inhibiting aptamer BT200. The difference between BT200 concentration needed to inhibit ristocetin induced aggregation to <20U was not significant before and after placebo (p=0.43). Baseline (0µg/ml) is depicted as 0.01 to improve visualisation in all figures with log scales.


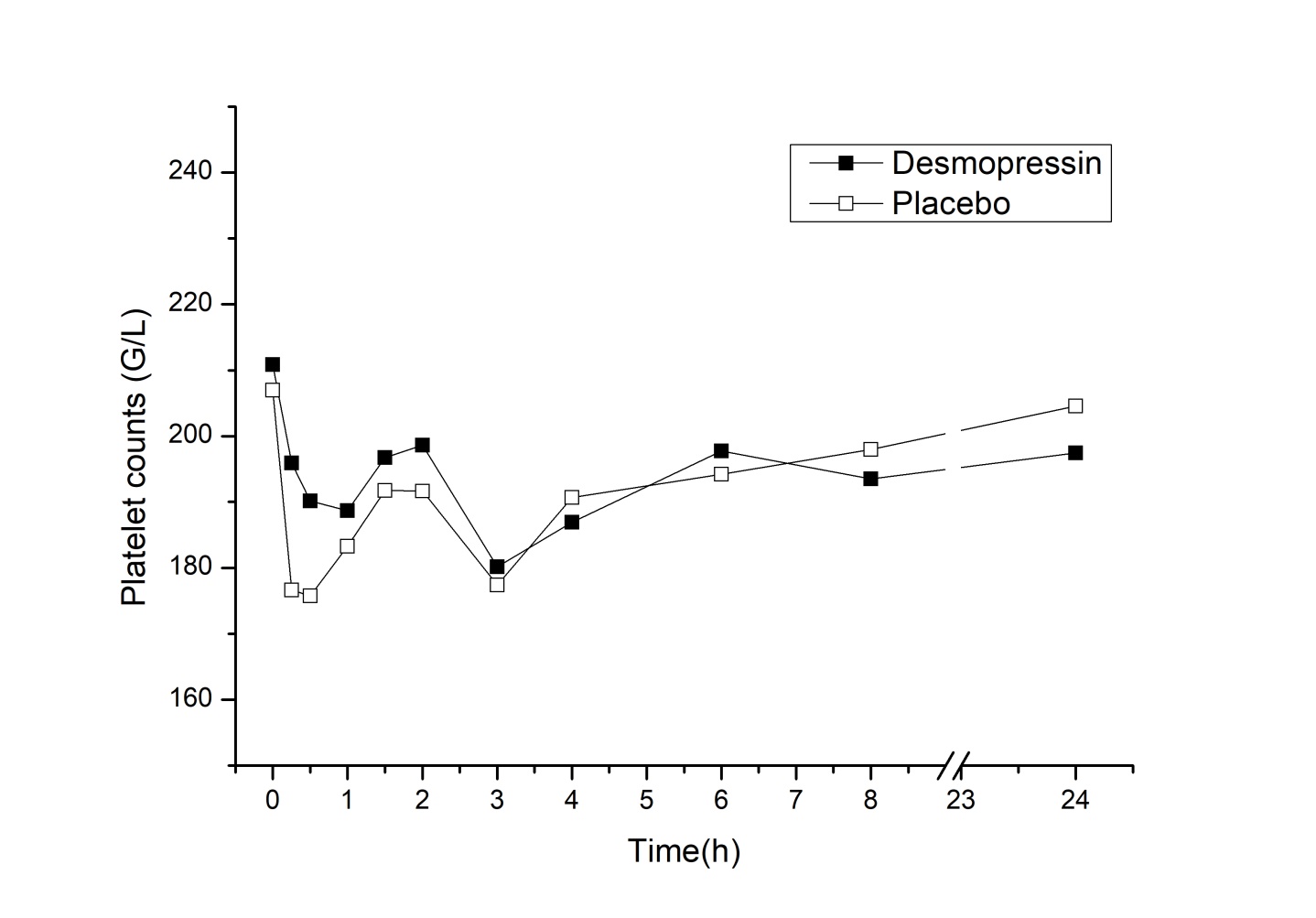


Supplementary Figure S6. Mean values of platelet counts after desmopressin and after placebo.


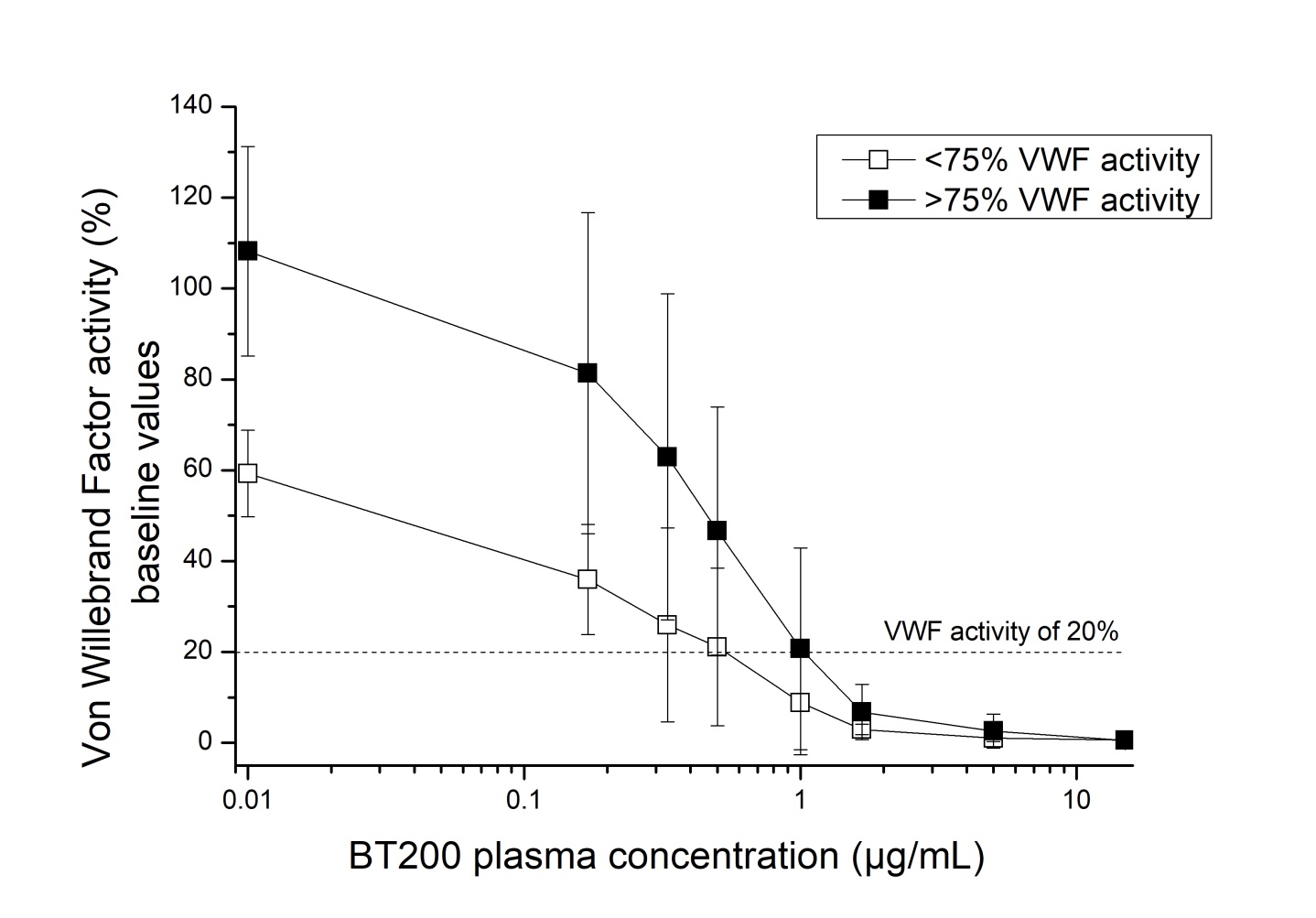


Supplementary Figure S7. Mean concentration-effect curve the BT200 on VWF activity plasma samples obtained from 38 healthy volunteers included in desmopressin or endotoxin studies before they received any treatment. Individuals were divided in two groups according to their VWF activity levels (<75% and >75%). Eight different BT200 concentrations were spiked into citrated plasma. The difference between BT200 concentrations needed to supress VWF activity to <20% of normal was significant between these two groups (p<0.001). Baseline (0µg/ml) is depicted as 0.01 to improve visualisation in all figures with log scales. (VWF-Von Willebrand Factor)


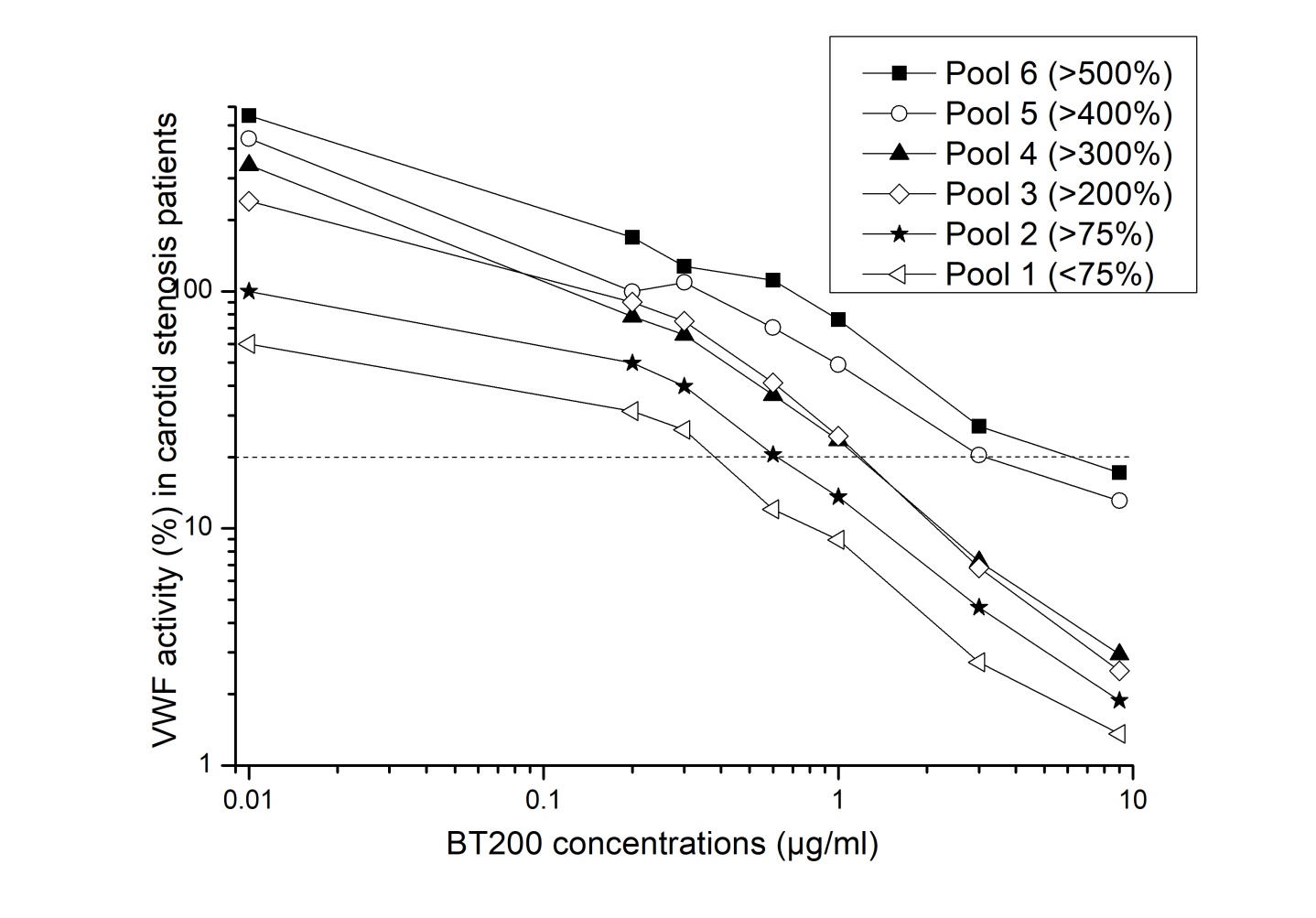


Supplementary Figure S8. Mean concentration-effect curve of BT200 on VWF activity in plasma samples obtained from 30 patients suffering from carotid stenosis. Individuals were stratified into 6 pools according to their VWF activity levels. Eight different BT200 concentrations were spiked into citrated plasma. Baseline (0µg/ml) is depicted as 0.01 to improve visualisation in all figures with log scales. (VWF-Von Willebrand Factor)


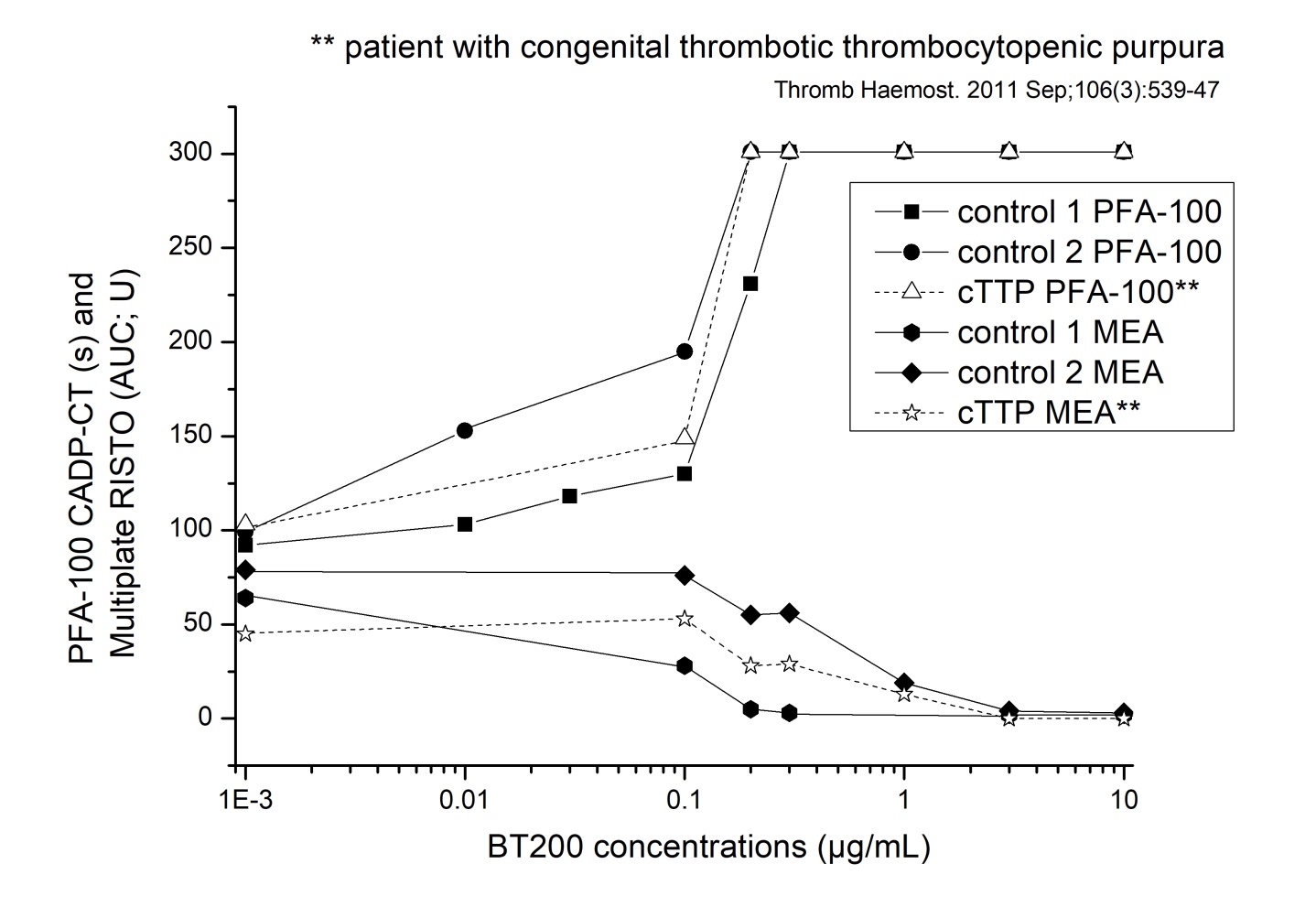


Supplementary Figure S9. Concentration-effect curve of BT200 on platelet plug formation under high shear rates (PFA-100) and on the ristocetin induced aggregation (MEA) in 2 healthy controls and a patient with congenital thrombotic thrombocytopenic purpura patients. Baseline (0µg/ml) is depicted as 0.01 to improve visualisation in all figures with log scales.
